# Necrotizing enterocolitis: a potential protective role for intestinal alkaline phosphatase as lipopolysaccharide detoxifying enzyme

**DOI:** 10.3389/fped.2024.1401090

**Published:** 2024-04-30

**Authors:** Raquel Dos Santos Martins, Jan B. F. Hulscher, Albert Timmer, Elisabeth M. W. Kooi, Klaas Poelstra

**Affiliations:** ^1^ Division of Pediatric Surgery, Department of Surgery, University of Groningen, University Medical Center Groningen, Groningen, Netherlands; ^2^ Department of Pathology and Medical Biology, University of Groningen, University Medical Center Groningen, Groningen, Netherlands; ^3^ Division of Neonatology, Department of Pediatrics, Beatrix Children’s Hospital, University of Groningen, University Medical Center Groningen, Groningen, Netherlands; ^4^ Department of Nanomedicine and Drug Targeting, Groningen Research Institute of Pharmacy (GRIP), University of Groningen, Groningen, Netherlands

**Keywords:** necrotizing enterocolitis (NEC), Toll-Like Receptor 4 (TLR4), lipopolysaccharide (LPS), intestinal alkaline phosphatase (IAP), enzymatic activity, prematurity and low birth weight

## Abstract

**Introduction:**

Necrotizing enterocolitis (NEC) is a life-threatening inflammatory disease. Its onset might be triggered by Toll-Like Receptor 4 (TLR4) activation via bacterial lipopolysaccharide (LPS). We hypothesize that a deficiency of intestinal alkaline phosphatase (IAP), an enzyme secreted by enterocytes that dephosphorylates LPS, may contribute to NEC development.

**Methods:**

In this prospective pilot study, we analyzed intestinal resection specimens from surgical NEC patients, and from patients undergoing Roux-Y reconstruction for hepatobiliary disease as controls. We assessed IAP activity via enzymatic stainings and assays and explored IAP and TLR4 co-localization through immunofluorescence.

**Results:**

The study population consisted of five NEC patients (two Bell's stage IIb and three-stage IIIb, median (IQR) gestational age 25 (24–28) weeks, postmenstrual age at diagnosis 28 (26–31) weeks) and 11 controls (unknown age). There was significantly lower IAP staining in NEC resection specimens [49 (41–50) U/g of protein] compared to controls [115 (76–144), *P* = 0.03]. LPS-dephosphorylating activity was also lower in NEC patients [0.06 (0–0.1)] than in controls [0.3 (0.2–0.5), *P* = 0.003]. Furthermore, we observed colocalization of IAP and TLR4 in NEC resection specimens.

**Conclusion:**

This study suggests a significantly lower IAP level in resection specimens of NEC patients compared to controls. This lower IAP activity suggests a potential role of IAP as a protective agent in the gut, which needs further confirmation in larger cohorts.

## Introduction

Necrotizing enterocolitis (NEC) is a life-threatening disease mainly affecting preterm infants. Mortality rates are up to 40%. Although the pathophysiology of NEC is yet not fully elucidated, abnormal intestinal bacterial colonization and immature gut immune responses are considered to play an important role (1). Treatment remains mainly supportive due to the lack of proper understanding of the pathophysiological mechanisms underlying NEC. Therefore, it is important to further investigate NEC pathophysiology to find new ways of targeted prevention or treatment (2–4).

Intestinal alkaline phosphatase (IAP) is an enzyme present in the apical membrane of intestinal epithelial cells and dephosphorylates several substrates such as lipopolysaccharide (LPS) (5–7). LPS is a characteristic component of gram-negative bacterial cell walls. The phosphate groups of LPS are recognized by Toll-Like Receptor 4 (TLR4), a pattern recognition receptor also expressed in intestinal cells (8). Upon activation by LPS, TLR4 signaling initiates intracellular pathways that activate pro-inflammatory and apoptotic transcription factors responsible for inflammation and cell death (8). When IAP dephosphorylates LPS, LPS undergoes neutralization, rendering it incapable of activating TLR4. Consequently, this process prevents further activation of TLR4 signaling (5–7).

Research has consistently shown that enteral administration of IAP in rat NEC pup models leads to a reduction in intestinal injury, a result corroborated by subsequent studies (9–14). While previous investigations have explored IAP activity in various biological samples, including blood, feces, and breast milk (15–17), there remains a gap in understanding the expression and actual enzyme activity of IAP in intestinal tissue from NEC patients compared to control tissue. Moreover, conventional IAP assays in NEC studies often utilize substrates like pNPP and high pH levels (i.e., pH 9.5) to measure IAP activity (15, 16, 18), leaving the detoxifying capacity of the intestinal wall in NEC patients against LPS at physiological pH levels (i.e., around pH 7) largely unexplored.

Highlighting the novelty of our study, our first aim was to investigate the differences in IAP dephosphorylating activity in intestinal samples between NEC patients and controls under physiological pH conditions. To our knowledge, this is the first study to directly compare the intestinal IAP enzymatic activity in NEC patients to that in patients without NEC, particularly at physiological pH levels. Based on previous findings and our observations (5, 6), we hypothesized that NEC patients would exhibit lower intestinal IAP enzymatic activity compared to patients without NEC. This investigation seeks to elucidate the potential role of diminished IAP detoxifying activity in contributing to the severe inflammation observed in NEC patients.

TLR4 expression in human tissue fluctuates between the cytoplasm and the cell membrane (19, 20). Colocalization of IAP and TLR4 in the intestinal epithelium would support an interactive role of both proteins in LPS-mediated inflammation (21). Therefore, the second aim of this study was to investigate whether there is a difference between IAP and TLR4 localization between intestinal samples from NEC and control patients. We hypothesized that the localization of TLR4 and IAP is different in intestinal specimens of NEC patients relative to controls.

## Methods

### Study population

This study included resection specimens from neonates born <30 weeks and/or <1,000 grams who developed surgical NEC and were operated between April 2021 and February 2023. All NEC patients were included in a prospective observational cross-sectional study (2019235 in the Dutch trial registry, METc 2019/235). After informed consent, neonates born <30 weeks and/or <1,000 grams were prospectively enrolled. For the present study, we included all consecutive infants who developed surgical NEC. Of these infants, we collected information on sex, gestational age (GA), birth weight (BW), postmenstrual age (PMA) at NEC diagnosis and surgery, delivery type, and exposure to antibiotics and probiotics as well as the use of mother's own milk (MOM), donor milk (DM), and formula feeding.

During November 2019 and February 2023, we also prospectively collected remnant jejunal and duodenal tissue from patients who underwent resection with Roux-Y reconstruction for hepatobiliary disease in our hospital. Therefore, our control cohort included resection specimens from patients of all ages from the neonatal period (i.e., Kasai procedures for biliary atresia) to late adulthood. As these resection specimens constituted waste material and were handled completely anonymously, no additional ethical approval was needed, apart from the general consent at the time of admission for the use of anonymous waste material for research purposes. This implies that we also did not collect any personal or medical data regarding these patients.

In our study, we utilized methods that required tissue storage at freezing temperatures rather than fixation with 4% formalin. Due to stringent regulations in the Netherlands regarding the collection of fetal tissue, no fetal tissue is stored at freezing temperatures following autopsy. As a result, our control group consisted of intestinal resections from patients undergoing Roux-Y reconstruction, who were generally older than those in the NEC group. Nonetheless, we made efforts to include age-matched specimens in the control group when employing immunofluorescence techniques. To achieve this, we included paraffin-embedded fetal tissue from cases at 26 weeks' gestation without gastrointestinal disease as controls (UMCG registry number 201800867). Clinical information was limited to gestational age and autopsy details. No informed consent was obtained for the leftover tissue used, and ethical approval was not mandatory in the Netherlands (METc 2019/263). However, we followed the Research Code of the University Medical Center Groningen and national ethical guidelines, submitting the study to the Central Ethical Committee for approval, which was granted (CTc letter 201800867).

### Definitions

NEC was defined as the presence of pneumatosis and/or portal air on abdominal x-ray, or during surgery. The pathology report was used to confirm the diagnosis when resection was performed. The intestinal resection specimen of all cases was reviewed blindly by an independent expert pediatric gastrointestinal pathologist (AT). Patients with spontaneous intestinal perforation (SIP) were excluded.

We defined postmenstrual age at diagnosis as the postmenstrual age of the patient when first showing pneumatosis intestinalis on X-BOZ, and age at surgery as the postmenstrual age of the patient at the date of surgery.

We calculated % of days the infants received MOM, DM, and/or formula feeding up until surgery by dividing the number of days in which neonates received any of the preparations by the total number of days of NICU stay until and including the day before surgery and multiplied by 100 to obtain percentages. If the neonate received more than one of the preparations, we counted each type of feeding as part of the whole. Halfway through the cohort study, we introduced DM and probiotics in our NICU for all infants born <30 weeks of gestation.

### Sample collection

We collected control and NEC intestinal resection specimens during abdominal surgery. We processed them in triplicate, using one sample for each of the following three methods (when possible/enough tissue): (1) standard alkaline phosphatase activity assay, (2) enzymatic staining for LPS dephosphorylation, and (3) immunofluorescence staining. The first sample was frozen in dry ice and stored in the −80°C freezer. The second sample was used for cryosections and therefore was embedded in tissue-tek (Sakura Finetek, Alphen aan den Rijn, The Netherlands) and stored in the −80°C freezer until 5µm sections were cut. Finally, the third sample was stored in 4% formalin for 24h at 4°C and thereafter submitted to an alcohol dehydration series and xylene before being embedded in paraffin. Paraffin blocks were stored in the −20°C freezer until 4µm sections were cut. To avoid completely necrotic areas, we analyzed the resection margins, which would be the closest to healthy tissue in NEC patients.

### Sample analysis

Enzyme activity was assessed using *p*-Nitrophenyl Phosphate (pNPP) assays, the BCIP/NBT AP-substrate kit, LPS-dephosphorylation staining, and staining quantification. Furthermore, we used double immunofluorescence to investigate the staining overlapped (i.e., colocalization) of IAP and TLR4. These techniques are fully described in the Supplementary Material.

### Statistics

Data were described as median and interquartile range (IQRs), or minimum-maximum, depending on the availability of data. Differences between the two groups were assessed using the Mann-Whitney *U* Test. All statistics were performed using IBM SPSS Statistics Version 28.0 (Armonk, NY, USA: IBM Corp.) and differences were considered statistically significant at *p* < 0.05.

We did not correct for potential confounders given the small sample size.

## Results

### Study population

At the time of inclusion, 122 patients were enrolled in the prospective cohort of which 11 patients had developed NEC. Of these 11 patients, six underwent surgery. Based on the pathology reports, five of these cases were confirmed as having NEC and these five therefore constituted our NEC cohort. The sixth turned out to have SIP. As controls, we included nine intestinal resection specimens from control patients of unknown age and fetal ileal resection specimens of two patients with GA of 26 weeks at the time of intrauterine death.

**Figure 1:**
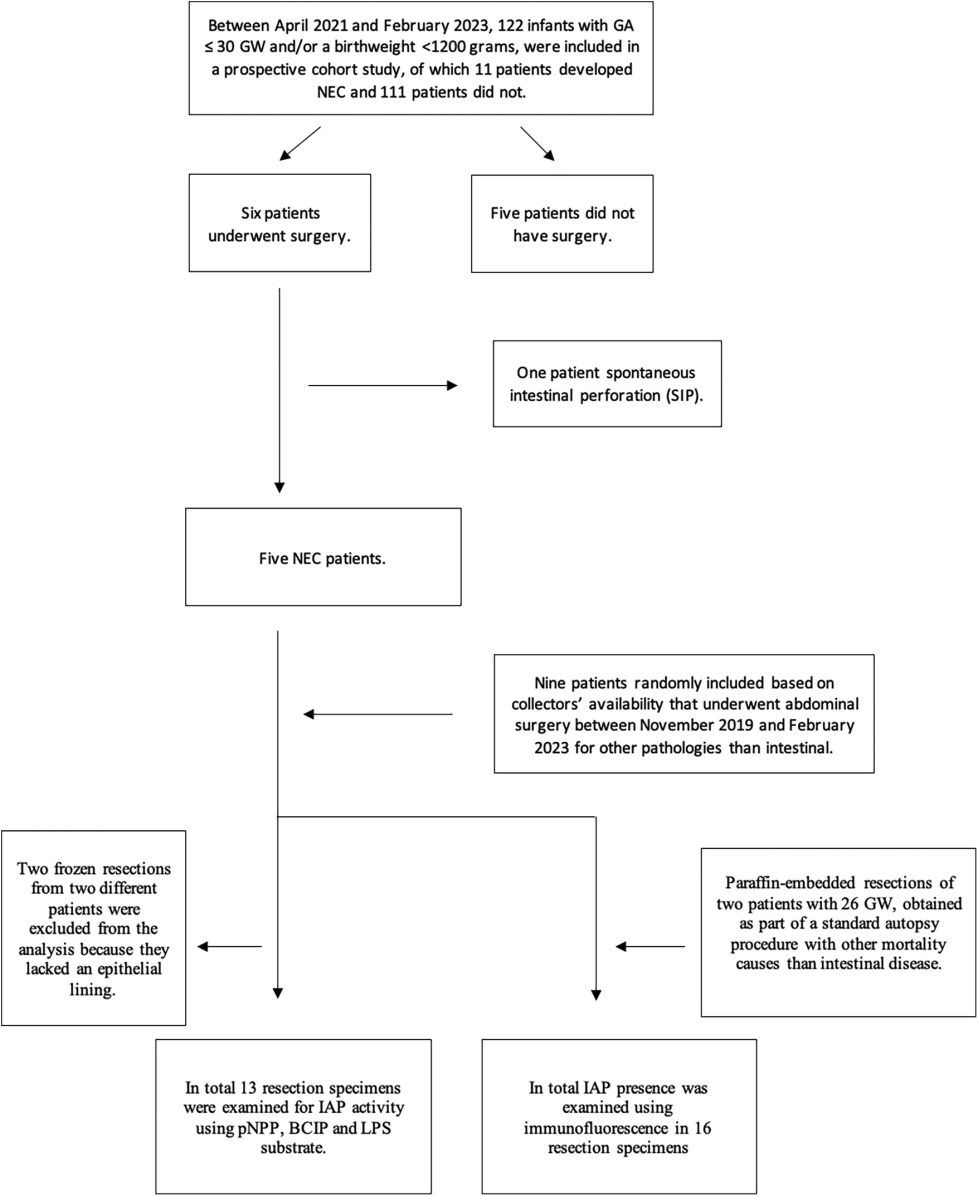
Inclusion flowchart.

A total of 13 resection specimens could be analyzed for IAP activity using pNPP, BCIP, and LPS substrate, and 16 resection specimens were used for immunofluorescence (as shown in Figure 1). Two frozen samples did not contain epithelial lining: one NEC intestinal resection specimen was not available for IAP activity assay using pNPP, and one control intestinal resection specimen was not available for histochemical detection of IAP with BCIP/NBT and LPS.

The NEC population consisted of four boys and one girl with a median GA of 25 (24–28) weeks and a median BW of 750 (720–987) grams. One patient was delivered through vaginal birth and the others through cesarean section. NEC was diagnosed at a median PMA of 28 (26–30) weeks, and the patients underwent surgery at a median PMA of 28 (26–31) weeks. Out of the five patients, two patients had Bell's stage IIb and three patients had stage IIIb (see Table 1).

**Table 1:** Characteristics of the study population.

Demographic characteristics of our NEC cohort		
*N*	5	
Sex		
Boys	4 (80%)	
Girls	1 (20%)	
Bell's stage		
IIa	0 (0%)	
IIb	2 (40%)	
IIIa	0 (0%)	
IIIb	3 (60%)	
Median gestational age in weeks (IQR)	25 (24–28)	
Median Birth Weight in grams (IQR)	750 (720–987)	
Median postmenstrual age at diagnosis in weeks (IQR)	28 (26–31)	
Median postmenstrual age at surgery in weeks (IQR)	28 (26–31)	
Delivery		
Cesarean	4 (80%)	
Vaginal	1 (20%)	
Number of patients receiving probiotics	1 (20%)	
Number of patients receiving antibiotics	5 (100%)	
Number of patients having positive blood cultures within 2 days before surgery	2 (40%)	
Species	Staphylococcus epidermis	
	Staphylococcus epidermis	
Number of patients receiving TPN until surgery	5 (100%)	
Median % of days infants received TPN until surgery (IQR)^a^	38 (31–71) %	
Number of patients receiving mother’s own milk until surgery	5 (33%)	
Median % of days infant received mother’s own milk until surgery (IQR)^a^	29 (17–60) %	
Number of patients receiving donor milk until surgery	1 (20%)	
Median % of days infant received donor milk exposure until surgery^a^	45%	
Number of patients receiving formula until surgery	3 (60%)	
Median % of days infant received formula until surgery (minimum-maximum)^a^	8 (2–18) %	
Study population intestinal sections	*NEC cohort*	*Control cohort*
*N*	5 (31%)	11 (69%)
Intestinal tissue part		
Duodenum-mucosa	0 (0%)	2 (13%)
Jejunum	0 (0%)	7 (43%)
Ileum	5 (31%)	2 (13%)

TPN, total parental nutrition.

^a^ Total feeding days might add up to more than 100% due to the usage of more than one type of feeding per day.

All NEC patients received antibiotics shortly before surgery and two patients (40%) had positive blood cultures within two days before surgery (*Staphylococcus capitis* and *Staphylococcus epidermis*). Five patients (100%) received total parental nutrition (TPN), five patients (100%) received MOM, one patient (20%) received DM, and three (60%) patients received formula feeding. Probiotics had been administered to one NEC patient (20%), who was born after the first introduction of probiotics in our NICU (see Table 1).

Resection specimens stemming from NEC patients constituted ileum and tissue from control patients was a mix of duodenum, jejunum, and ileum (see Table 1).

### IAP activity

The enzymatic activity assay measuring the IAP ability to dephosphorylate pNPP in intestinal resection specimens showed a median (IQR) IAP activity in resection specimens of NEC patients of 49 (11–50) U/g of protein vs. 115 (26–211) U/g of protein in controls, *P* = 0.03 (see Table 2).

**Table 2:** IAP activity assay using pNPP in NEC and control patients.

	NEC cohort (*n* = 4)	Control cohort (*n* = 9)	*P* value
Median IAP activity assay (U/g of protein) (IQR)	49 (41–50)	115 (76–144)	**0** **.** **03** ^a^

^a^ Using the Mann-Whitney Test, a two-tailed test; Statistical significant results are presented in bold format.

IQR means interquartile range.

**Figure 2:**
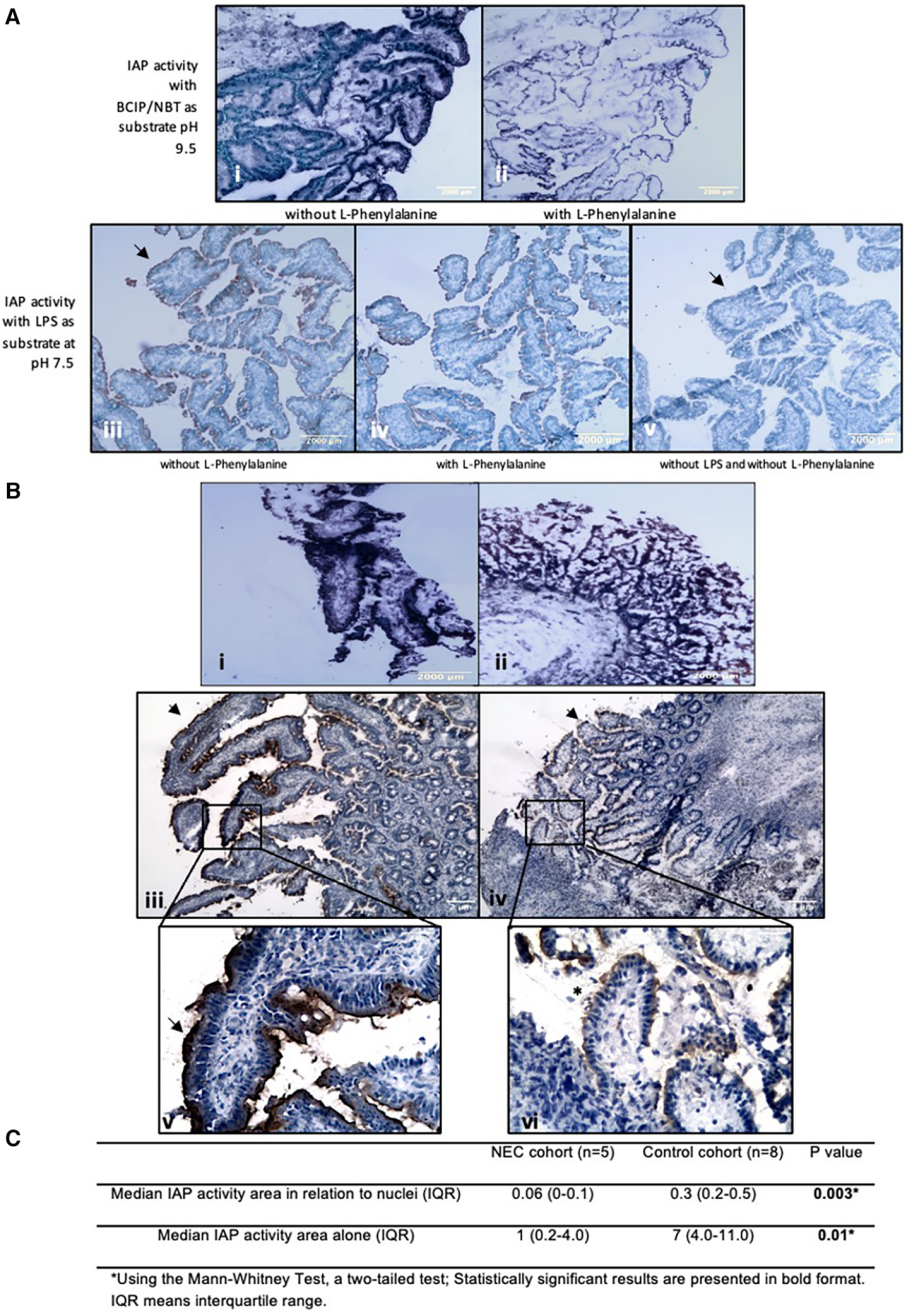
Histochemical detection of IAP activity intestinal resection specimens from control patients. LPS dephosphorylation by IAP is lower in intestinal resection specimens from NEC patients compared to control patients. (**A**) Histochemical detection of IAP activity in intestinal resection specimens from control patients. (i and ii) BCIP dephosphorylation by IAP in intestinal resection specimens from control patients (*n* = 8). (i) Incubation without L-Phenylalanine and (ii) with 100 mM of L-Phenylalanine as an inhibitor of IAP activity. Purple staining is visible along the intestinal microvilli apical side, which represents the dephosphorylation of BCIP by AP. (iii, iv, and v) LPS dephosphorylation by IAP in intestinal resection specimens from control patients (*n* = 5). (iii) Incubation with 2.5 mg/ml LPS, (iv) with 2.5 mg/ml LPS and 100 mM of L-Phenylalanine, (v) without LPS or inhibitor. Brown staining is visible along intestinal microvilli apical membrane in positive sections but not in negative sections (see arrowheads). Note that the staining for LPS dephosphorylation can be seen at the same localization as BCIP dephosphorylation by AP. Sections incubated with L-Phenylalanine an inhibitor of IAP show less staining. (Magnifications i and ii 100x, Magnifications iii, iv, v, 40X). (**B**) Histochemical detection of IAP activity in intestinal resection specimens from NEC patients. (i) Representative pictures of BCIP dephosphorylation by IAP in intestinal resection specimens from control patients (*n* = 8, 40x total magnification) and (ii) in intestinal resection specimens from NEC patients (*n* = 5, 40x total magnification). (iii and v) LPS dephosphorylation (brown/black lining, see arrowheads) by IAP in intestinal resection specimens from control patients (*n* = 8) and (iv, and vi) in intestinal resection specimens from NEC patients (*n* = 5). (iii and iv) 40x and (v and vi) 100x total magnifications. NEC patients’ intestinal resection specimens had less intense BCIP and LPS staining than control patients’ resections, showing that the intestinal IAP activity in these patients is lower. Note that in picture vi, areas with intact vili (*) displaying a lower or non-continuous (see arrowheads) LPS staining can be seen, indicating that low IAP is not induced by tissue destruction. Our results were consistent across the different samples. (**C**) Quantification of histochemical staining of LPS-dephosphorylation using image analysis.

In resection specimens from NEC patients, histochemical detection of IAP dephosphorylating activity using either BCIP or LPS as substrate showed less staining intensity when compared to resection specimens from controls (Figures 2A,B). This indicates lower IAP dephosphorylating activity in the intestine of NEC patients when compared to controls. This was not due to the occurrence of necrotic areas, as intact villi also displayed reduced or non-continuous LPS staining activity with LPS as substrate (see Figure 2B).

In resection specimens from control patients, histochemical studies demonstrated IAP dephosphorylating activity using BCIP as substrate in the apical membrane of the intestinal mucosal layer of controls (see Figure 2A). Similar results were obtained after the histochemical detection of LPS dephosphorylation by IAP (see Figure 2A). To demonstrate the specificity of stainings, control stainings were performed with the IAP-inhibitor L-phenylalanine (22) added to the incubation media, or substrates were omitted. The latter sections were devoid of any staining while L-phenylanine strongly inhibited staining in all cases, including the LPS-dephosphorylating activity. These results showed that there is dephosphorylation of LPS by IAP in the intestinal wall (see Figure 2A).

Image analysis determining the area of LPS-dephosphorylating activity in enterocytes relative to the total nuclear area in the section, which indicates the number of cells in the entire section, showed that ileum from NEC patients had a median (IQR) IAP activity area in relation to nuclei of 0.06 (0–0.1) per cell, while intestinal tissue from control patients had a median (IQR, *P* value) of 0.3 (0.2–0.7; *P* < 0.01) per cell. This was also the case when comparing the quantification of the area of LPS-dephosphorylating activity alone (i.e., without relation to the total nuclear area), which was 1 (0.02–5) in the ileum of NEC patients and 7 (3–11) in control tissue, *P* = 0.01 (see Figure 2C).

### IAP and TLR4 localization

**Figure 3:**
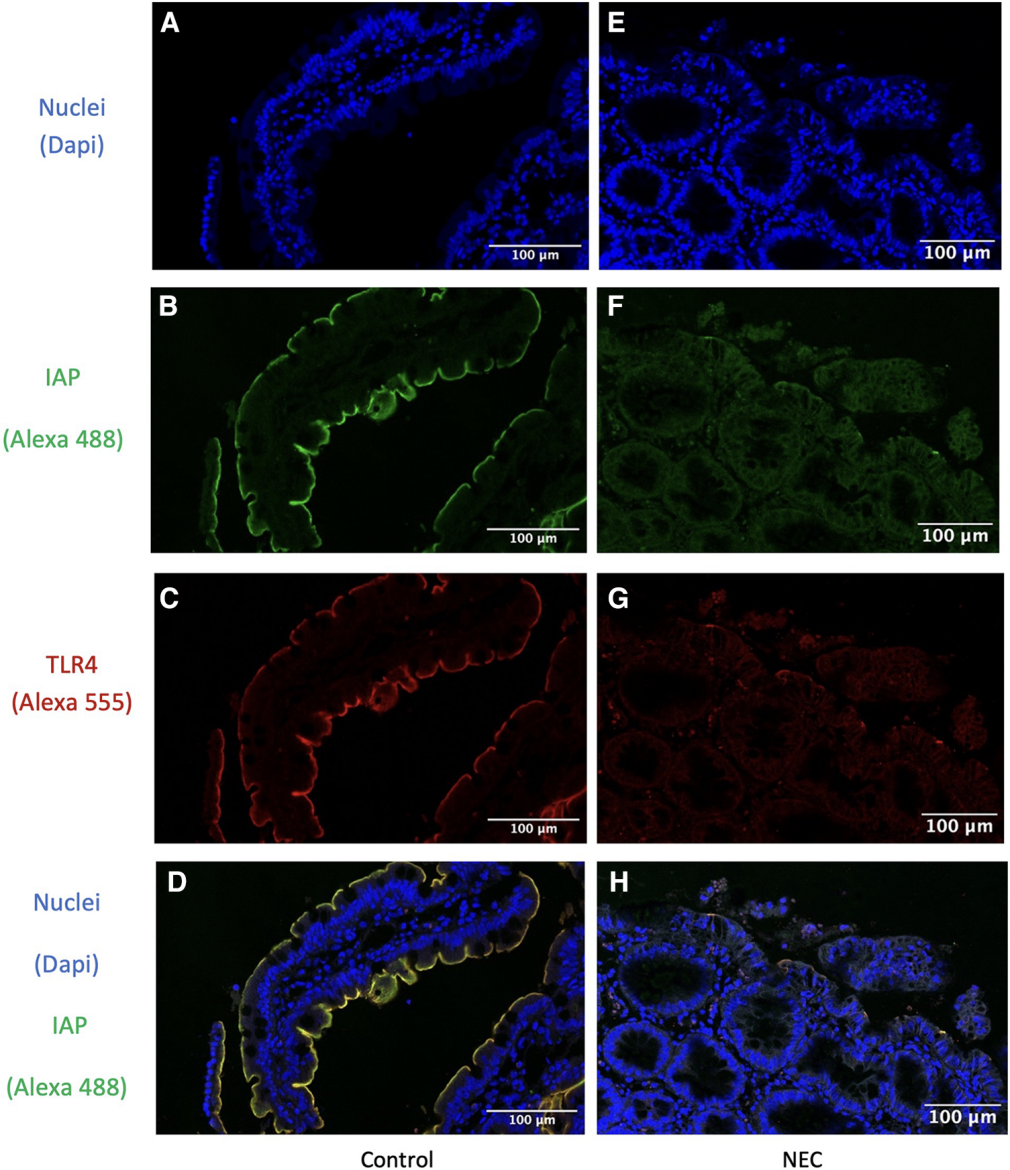
Immunofluorescence detection of IAP and TLR4 in intestinal resection specimens from representative control patients and in intestinal resection specimens from NEC patients. IAP is colocalized with TLR4 in both intestinal resection specimens from control and from NEC patients. (**A,E**) Dapi channel showing the enterocyte cell nuclear staining in blue in (**A**) intestinal resection specimens from control (*n* = 9) and (**B**) intestinal resection specimens from NEC patients (*n* = 5). 63x total magnification. (**B,F**) Alexa 488 channel showing the IAP staining in green in (**B**) intestinal resection specimens from control (*n* = 9) and (**F**) intestinal resection specimens from NEC patients (*n* = 5). 63x total magnification. (**C,G**) Alexa 555 channel showing the TLR4 staining in red in (**C**) intestinal resection specimens from control (*n* = 9) and (**G**) intestinal resection specimens from NEC patients (*n* = 5). 63x total magnification. (**D,H**) Overlay of the three channels showing the enterocytes cell nuclei staining in blue, the IAP staining in green, and the TLR4 staining in red, in (**D**) control samples (*n* = 9) and (**H**) NEC samples (*n* = 6). 63x total magnification. The yellow staining represents the co-localization of IAP and TLR4 colocalized. Furthermore, in IAP fluorescence staining is less intense in intestinal resection specimens from NEC patients compared to intestinal resection specimens from control patients, while TLR4 staining was lower in enterocyte's membranes but not in their cytoplasm. Our results were consistent across the different samples.

In all resection specimens from control patients, we observed expression of both membranous and cytoplasmic TLR4 (shown in red in Figure 3). IAP, on the other hand, was only detected at the apical side (shown in green in Figure 3). Membranous TLR4 expression but not cytoplasmic expression was lower in NEC cases compared to controls. The intensity of IAP staining was also weaker in NEC cases. Immunofluorescence analysis revealed overlapping staining of IAP and TLR4 in both intestinal specimens from NEC and control patients (depicted in yellow in Figure 3), indicating colocalization of these proteins.

### IAP expression in relation to anatomic location and age

**Figure 4:**
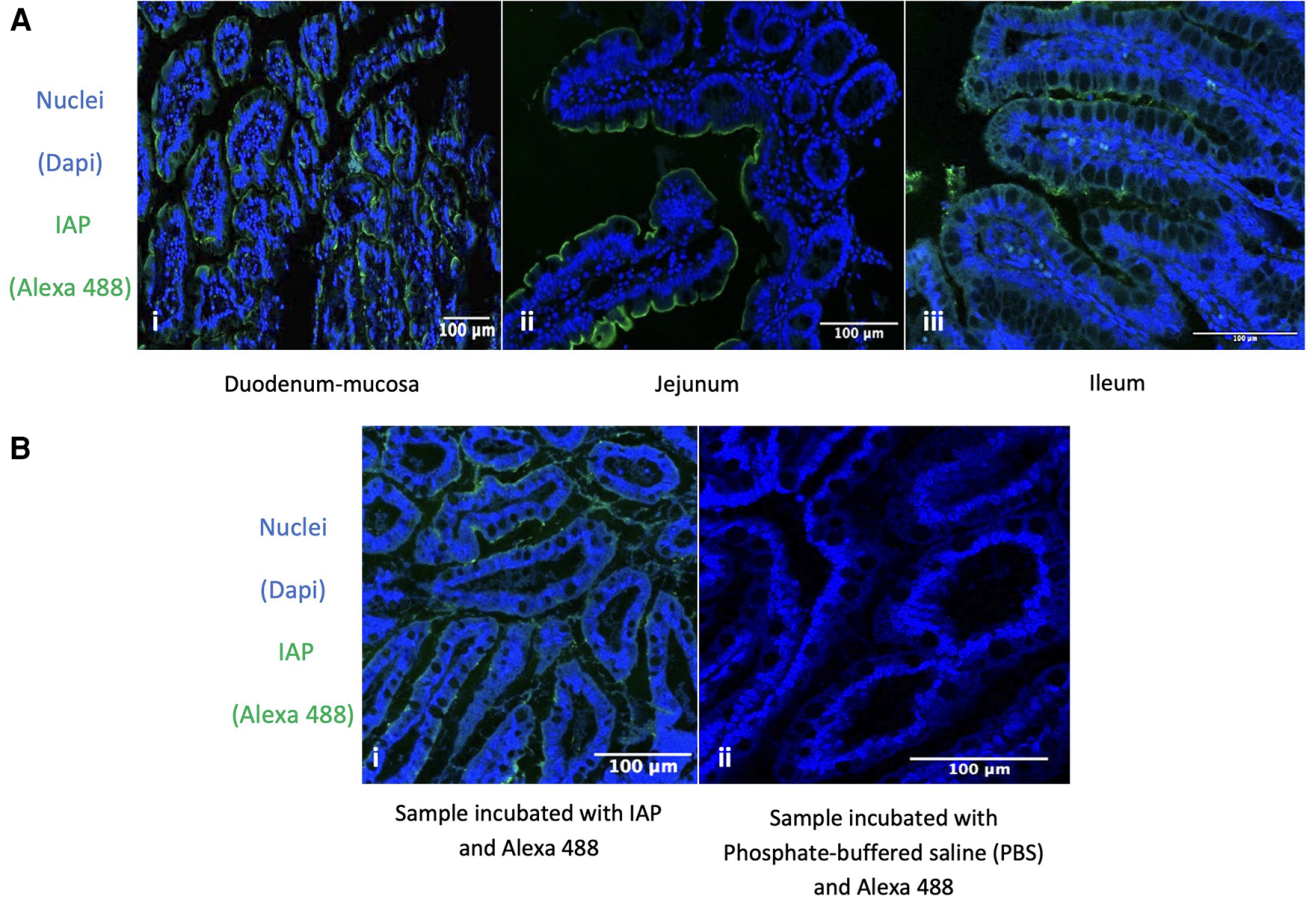
Immunofluorescence detection of IAP. Expression of IAP in the Duodenum, Jejunum, Ileum, and its Specific Expression in the Ileum of Fetuses at 26 Weeks Gestational Age. (**A**) *Immunofluorescence detection of IAP across different parts of the healthy small intestine.* (i, ii, iii) Intestinal nuclear staining (blue: DAPI) and IAP staining (green: Alexa 488) in different sections of the intestine. (i) Human healthy duodenum-mucosa, *n* = 2. (ii) Human healthy jejunum, *n* = 7. (iii) Human healthy ileum, *n* = 2. 63x total magnification. Our results were consistent across the different samples. (**B**) *Immunofluorescence detection of IAP in ileum resection specimens from 26 GW fetus.* (i, ii) Nuclear staining (blue: DAPI) and IAP staining (green: Alexa 488) show the nuclear staining in blue (i, ii) and IAP expression in green (i). (ii) Only the secondary antibody was used, showing no staining at all. 63x total magnification and *n* = 2. All our results were homogenous across the different samples. The specific green staining at the enterocytes’ apical membrane indicates that IAP is expressed in the fetus's ileum at the age of 26 GW.

To rule out differences between duodenum, jejunum, and ileum we studied the expression of IAP (using immunofluorescence) in all these areas in control patients and did not find any differences. Therefore, IAP appeared to be expressed throughout the healthy small intestine (Figure 4A).

Immunofluorescence detection of IAP in ileum resection specimens from human fetuses showed that IAP was expressed in the developing gut at 26 GW (see continuous staining in the enterocyte's apical membrane in Figure 4B).

## Discussion

In this prospective observational pilot study, we investigated how IAP activity in intestinal samples differs between NEC patients and controls. We observed that while IAP is still present in most of the resection specimens of NEC patients, its expression and dephosphorylating activity is significantly lower than in controls. Also, we found that IAP and TLR4 colocalize in the apical membrane of enterocytes of both control and NEC patients. Finally, we demonstrated that TLR4 expression in the apical membrane of enterocytes is lower in resection specimens stemming from NEC patients when compared to controls. Together this suggests that IAP activity is lower in NEC patients and that IAP and TLR4 colocalize in the apical membrane of NEC patients, suggesting an interactive role.

IAP was first linked to LPS-induced diseases by Poelstra et al., showing that IAP was able to dephosphorylate, and thus detoxify, LPS (6, 23). The group demonstrated subsequently that IAP could be used for the treatment of sepsis and other diseases induced by LPS in mice models (24–26). Whitehouse et al. showed that enteral administration of IAP to rat NEC pup models resulted in regression of intestinal injury, and this was later confirmed by other research groups (9–14). Since then, IAP activity has been studied in blood, feces, and breast milk (15–17). In blood, patients with spontaneous intestinal perforation had higher serum IAP activity upon admission than patients with NEC (17). In feces, patients with NEC had low IAP activity (15, 16). Finally, it was found that IAP activity in the mother's own milk (MOM) was high in the first week post-birth and followed by a progressive decline in activity in weeks two to seven post-birth (16). In our study, we measured the ability of IAP to dephosphorylate pNPP, BCIP, and LPS in NEC patients and in controls. In line with previous studies, we were able to observe that in NEC patients, there is less IAP activity in intestinal resection specimens when compared to controls. For the first time, we measured LPS detoxifying activity—and not just expression—in intestinal resection specimens obtained from NEC and control patients. As LPS dephosphorylation leads to the detoxification of bacterial products (6), the lower LPS-dephosphorylating activity we found in NEC patients, might be an important factor in the pathogenesis of NEC.

In our study, we assessed IAP activity in the intestinal wall. Although IAP can be found in serum and feces as well, these levels may not reflect the local enzyme activity in the intestinal wall. In addition, studying IAP in serum is challenging as there are several types of AP (i.e., IAP, tissue-nonspecific AP, and placental AP). These enzymes are encoded by different genes, but they have similar structures (27, 28). This makes studying specific AP serotypes separately difficult, as a selective inhibitor that completely blocks a specific isoenzyme is yet unavailable (29). Hence, IAP activity in the serum of NEC patients might not reflect the actual intestinal IAP activity. Likewise, patients' feces accommodate many distinct species of bacteria that can produce their own AP enzymes (29). Therefore, extrapolating IAP activity in the intestinal wall of NEC patients, based on AP activity in the serum or feces of NEC patients is less reliable. Also, traditional methods using high pH levels (i.e., 9–10) and chemical substrates to measure IAP activity, may not reflect physiological significance. Yet, histochemical detection of LPS dephosphorylation in intestinal resection specimens from NEC patients at physiologic pH (i.e., 7.5) allowed us to mimic the natural LPS detoxification process that occurs in the intestinal wall of NEC patients (pH of 7.4–8.0).

In our study, we found both a lower expression and activity of IAP in ileum resection specimens stemming from NEC when compared to specimens from control patients. One possible explanation for our findings is that structural alterations occur in IAP during intestinal development, in preterm infants compared to those born full-term, which may result in divergent structures that may not be recognized by current antibodies, as well as in differences in detoxification activities (30–35).

As many studies have indicated a role for bacteria and LPS in the pathogenesis of NEC, the LPS receptor TLR4 has been often studied in connection with this disease (8). Although little is known about its expression/localization in the human intestine, TLR4 expression is thought to fluctuate between the cytoplasm and the cell membrane (19, 20, 36). In the intestine of NEC patients, TLR4 expression remains an intricate topic (37). On one hand, Leaphart et al. demonstrated a significant increase in TLR4 expression in resections from both NEC patients and NEC experimental models (21). On the other hand, other studies have indicated a decrease in TLR4 expression in samples derived from NEC patients (38, 39). In our study, we observed a specific decrease in TLR4 expression in the apical membrane of enterocytes, without a corresponding decrease in the cytoplasm. However, we exercise caution in our interpretation due to the qualitative nature of our study and the limitations of immunofluorescence analysis, hindering quantitative conclusions. We acknowledge the challenges of comparing results obtained from different techniques. While a decrease in the apical membrane of enterocytes is evident, an increased expression in the cytoplasm of enterocytes may balance out these decreases when assessing TLR4 expression overall, quantitatively.

IAP and TLR4 colocalization has previously been investigated in intestinal resection specimens from patients with Crohn's disease. This study also found a co-localization between IAP and TLR4 (40). In the present paper, we demonstrate that TLR4 and IAP are colocalized in resection specimens from both NEC and control patients. This suggests a functional connection between both proteins. Although we expected to see IAP and TLR4 colocalized in intestinal resection specimens from NEC patients, we did not expect that this would also be the case in intestinal resection specimens from control patients. The fact that TLR4 is expressed at the apical plasma membrane of normal enterocytes is a surprise as its constitutional expression would elicit an inflammatory process in the presence of LPS. As the intestinal lumen is in general full of LPS but our control group had no ongoing inflammation, it can be hypothesized that a mechanism suppresses TLR4 responses upon stimulation by this bacterial product.

One plausible explanation for the unexpected colocalization of TLR4 and IAP in control ileum resection specimens, as well as the lower expression of TLR4 in the enterocyte membrane seen in intestinal resection specimens stemming from NEC patients, is that TLR4 and IAP are consistently co-localized in the apical membrane of enterocytes. In this scenario, IAP plays a protective role by preventing the activation of TLR4 by LPS. However, in the absence of IAP and the presence of inflammation, TLR4 may become activated and subsequently translocate to the cytoplasm (41). As intestinal resection specimens are not always collected under the same conditions, variations in TLR4 expression and localization may be observed.

### Limitations

This study has several limitations. First, it is important to note that obtaining tissue from a control group matching the NEC cohort in terms of age, feeding, and intestinal section is virtually impossible. Consequently, in our study, the NEC and control populations were not matched for these factors, potentially confounding our findings. The NEC population had a median PMA at surgery of 28 weeks, while the control population included patients ranging from the neonatal period to late adulthood. However, in intestinal resection specimens from autopsies with approximately the same GA as NEC cases (26 weeks' gestation), we observed widespread expression of IAP in the ileum. We argue that the observed differences between the two patient populations are therefore unlikely to be solely attributed to age differences, and we consider it improbable that our results can be explained by preterm infants having lower IAP expression than full-term children. Nevertheless, certainty in this regard cannot be guaranteed. Second, our NEC cohort primarily consisted of ileum resection specimens, while the control cohort included duodenum and jejunum tissue. We demonstrated that IAP is typically present throughout the healthy small intestine in all three parts: duodenum, jejunum, and ileum (see Figure 4A) suggesting that the difference in anatomical location of the samples is not the cause of the variation in IAP. This supports the notion that the lower AP activity found in NEC patients compared to control samples is not due to the selection of different parts of the small intestine. Third, although diet, specifically fat intake, influences IAP expression, this factor is unlikely to play a role here since all patients in both groups were not fed before obtention of the intestinal resection specimen, as patients need to fast before surgery with general anesthesia and NEC patients are on a nil per os regimen per definition (42). In addition, both patient groups, i.e., NEC and control patients, comprised all other types of feeding, while the results were consistent in all resection specimens. Lastly, the differences in the activity of IAP in specimens from NEC vs. control patients could theoretically be explained by an abnormally increased prevalence of bacteria in the intestine of NEC patients compared to control. In this scenario, IAP activity could be “overwhelmed” by the amount of LPS present in the intestinal wall of these patients. This could indeed explain the decrease in IAP activity, as measured in the resection specimens from NEC patients. However, one would also expect to observe maintained IAP protein expression in these ileum resection specimens. This was not the case, as we observed both reduced IAP activity against LPS and decreased IAP expression. Enzyme activity can be regulated by the availability of the enzyme's substrate, but enzyme expression is not. Therefore, this suggests an overall absence of IAP in the ileum of NEC patients, which cannot be solely attributed to an excess of LPS that surpasses the enzyme's detoxification capacity.

On top of the already mentioned limitations, we recognize that our NEC cohort included only five NEC patients. Also, ileum resection specimens derived from patients are partly necrotic and necrosis is a process associated with loss of epithelial cells (43). Yet, intact areas with little or no IAP activity support the argument that these patients have lower activity of IAP than normal, which is not due to tissue destruction or inflammation. Interestingly, in animal models, ischemic colitis is associated with an increase in IAP, which contrasts with our findings (44). Similarly, myocardial infarction has been shown to result in elevated IAP levels (45). These observations suggest that ischemia itself may not be the primary cause of the decreased IAP levels observed in our samples.

Given the multiple limitations of this study, it is imperative that our experiments be replicated in future studies using larger, age-matched cohorts. Ideally, these studies should utilize frozen ileum resection specimens from both NEC patients and control subjects (e.g., fetal tissue obtained from abortion clinics)”.

## Conclusion

In NEC patients, ileal levels of IAP protein were present but lower than in controls. Furthermore, LPS-detoxification activity, a key function of IAP, was also lower in NEC patients compared to controls. Interestingly, IAP and TLR4 co-localized in enterocyte apical membranes, suggesting a potential functional interaction between these proteins, which aligns with the known role of IAP as an LPS detoxifying enzyme. These findings support previous studies highlighting the importance of investigating IAP's role in NEC pathophysiology.

Notably, our study was limited by the inability to identify a matched control cohort due to ethical constraints. Future studies should address this limitation by including controls matched for both intestinal segment and GA, enhancing the generalizability and strength of the conclusions.

## Data Availability

The original contributions presented in the study are included in the article/Supplementary Material, further inquiries can be directed to the corresponding author.
