# Supplementary material for: Necrotizing enterocolitis: a potential protective role for intestinal alkaline phosphatase as lipopolysaccharide detoxifying enzyme
Source: Front Pediatr. 2024 Apr 30;12:1401090. doi: 10.3389/fped.2024.1401090 (PMC11091495; doi:10.3389/fped.2024.1401090)
Supplement: Supplementary file 1 [file Datasheet1.pdf]

Supplementary Material

# Methods

**AP activity assay using p-Nitrophenyl Phosphate (pNPP) at pH 9.8.**

1. Control and NEC patients' samples preparation.

Intestinal resection specimens stored at -80°C were unfrozen in the incubator at 37°C for five minutes. Next, the resection specimen was washed two times with deionized water, using a quick centrifugation program, and thereafter it was homogenized, also with deionized water (1:1 volume ratio), for 15 minutes. Subsequently, the water-resection mix was centrifuged at 10°C for 10 minutes at 15000g. The supernatant was divided into two tubes: one tube was used for the activity assay and the other for the determination of total protein content.

2. Activity assay.

An IAP activity assay was conducted using the conventional IAP substrate p-Nitrophenyl Phosphate (pNPP). The supernatant was further diluted with solution buffer (pH 9.8) which contained 2-amino-2-methyl-1,3-propanediol (1M, Sigma, St Louis, Missouri, USA) and $MgCl_2$ (0.5mM, Boom BV, Meppel, NL) applying 1/100, 1/500, and 1/1000 dilutions, to ensure at least two readings within the linear range of the standard curve. PNPP (Sigma, St Louis, Missouri, USA) was added at t=0 (final concentration 5 mM). Reference curves were created using the enzymatic end product of pNPP, p-nitrophenol (pNP, 1mM, Merck KGaA, Darmstadt, Germany) in the following concentrations: 0, 0.025, 0.05, 0.075, 0.1 and 0.125 mmol per l buffer. Each sample was measured in triplicate. All samples were incubated in the microplate reader (Molecular devices ThermoMax) at 37°C for 30 min and O.D values were recorded at 405 nm and enzyme kinetics were analyzed. Enzyme activity was expressed in enzyme units (U, µmol/min) using the reference curves. All data points are the average of three experiments.

3. Protein quantification assay.

The second tube was used for a protein quantification assay using the Pierce™ BCA protein assay kit (Fisher Scientific Thermo Scientific™) in triplicate with bovine serum albumin

(BSA) as reference protein according to standard methods and using a microplate reader. Protein concentration (mg/ml) was related to enzymatic activity, yielding U/g protein.

**Histochemical detection of IAP activity with 5-bromo-4-chloro-3-indolyl phosphate/nitroblue tetrazolium (BCIP/NBT) at pH 9.5.**

We used the BCIP/NBT substrate kit (Vector laboratories, Newark, California, USA) to stain for IAP activity in intestinal cryo specimens. Briefly, two sections per patient were fixed in 4% formalin-macrodex-$CaCl_2$ for 10 minutes at 4°C. After washing, sections were incubated with the substrate in Tris-HCl buffer (0.1M, Promega Corporation, WI 53711 USA) at pH 9.8, for 30 minutes at room temperature. Paired sections were incubated either with or without L-phenylalanine (100mM, Sigma, St Louis, Missouri, USA), a known inhibitor of AP. [44,45] Sections were analyzed using an Olympus BX50 microscope (Olympus, Tokyo, Japan).

**Histochemical detection of LPS-dephosphorylation at pH 7.5.**

1. Staining

LPS-dephosphorylating IAP activity was stained in intestinal cryo sections using LPS from Escherichia coli (2.5 mg/mL, serotype O55:B5, Sigma, St Louis, Missouri, USA), as substrate as described before [22]. Shortly, three sections per patient were fixed in 4% formalin-macrodex-$CaCl_2$ for 10 min at 4°C. After washing, sections were incubated at 37°C for 1 hour. One section was incubated with only buffer at pH 7.5 (control), one with LPS in buffer, and one with LPS, plus L- phenylalanine (100mM, Sigma, St Louis, Missouri, USA) in the buffer. The buffer contained Tris-HCl buffer (0.2M, Promega Corporation, WI 53711 USA), $MgSO_4$ (0.02M, Sigma, St Louis, Missouri, USA), and $Pb(NO_3)_2$ (5 mg/mL, Merck KGaA, Darmstadt, Germany) at pH 7.5. The method is based on the visualization of enzymatic phosphate release using $Pb^{2+}$, which causes a localized lead—phosphate precipitate. This is subsequently converted to lead sulfate by incubation with $Na_2S$, yielding brown staining. Sections were counterstained with Haematoxylin according to standard methods and examined with an Olympus BX50 microscope (Olympus, Tokyo, Japan).

2. Staining quantification.

To quantify the IAP dephosphorylating activity of LPS, we measured LPS dephosphorylation staining, obtained by histochemical detection of LPS-dephosphorylation at pH 7.5. We used

Fiji software (Fiji is Just ImageJ, version 2.9.0/1.53t, Open-source image processing software, copyright 2010-2022) color deconvolution setting to measure separately both IAP dephosphorylating activity staining and nuclear staining areas. Nuclear staining was used to assess the total number of cells in the section. Final values were presented as the ratio between the total area of IAP dephosphorylating activity staining and the total area of the nuclear staining. [46]

**Immunofluorescence detection of IAP and TLR4 in intestinal tissue from fetuses, control, and NEC patients.**

Two sections per patient underwent deparaffination and antigen retrieval overnight with Tris-HCl (0.1M, Promega Corporation, WI 53711 USA) at 80°C. Next, sections were blocked with 5% goat serum for 30 minutes at room temperature, and one section per patient was stained with both primary antibodies against IAP (1/500, ab186422, Abcam, Cambridge, UK [47]) and TLR4 (1/200, ab13556, Abcam, Cambridge, UK [48-50]). As our primary antibodies were from the same animal species, we coupled TLR4 with Alexa 555 (Invitrogen, Waltham, Massachusetts, USA), according to standard methods. Briefly, 10 µl TLR4 antibody and 5 µl of Alexa 555 label were mixed in 10 µl bicarbonate buffer (0.2M, pH=8.3, Merck KGaA, Darmstadt, Germany) for 1 hour at room temperature. The unbound label was removed with PBS by centrifuging this mix in a 3,000 Dalton filter (Da, Pall Corporation, New York, United States), at 5,000g for 10 min, which was repeated at least five times. First, IAP staining was detected using Alexa 488-labeled GaR (1/200, Invitrogen, Waltham, Massachusetts, USA) and then the labeled TLR4 antibody was used to stain for TLR4 to avoid crossed recognition. Control sections were only stained with both secondary antibodies, 488GaR (1/200, Invitrogen, Waltham, Massachusetts, USA) and 555GaR (1/200, Invitrogen, Waltham, Massachusetts, USA). We examined fluorescence staining using a Leica TCS SP8 X confocal microscope (© 2022 Leica Microsystems).

Supplementary Table 1– Raw Data Analysis of IAP activity.

| | NEC cohort (n=5) | | | | | Control cohort (n=9) | | | | | | | | |
|---|---|---|---|---|---|---|---|---|---|---|---|---|---|---|
| Patient ID | 1 | 3 | 5 | 13 | 14 | 7 | 8 | 9 | 10 | 11 | 12 | 15 | 16 | 17 |
| IAP activity assay (U/g of protein) | 39,04 | 49,97 | 50,05 | NA | 48,88 | 103,63 | 143,78 | 211,69 | 115,63 | 145,00 | 97,52 | 26,4 | 118,84 | 55,37 |
| IAP activity area in relation to nuclei x $10^2$ | 0,09323 | 1,31536 | 6,09199 | 14,34785 | 14,48885 | 18,45309 | 30,06642 | 41,56777 | 37,74212 | 22,89683 | 19,47964 | 52,78007 | NA | 66,97064 |
| IAP activity area alone | 0,021 | 0,321 | 1,106 | 4,915 | 3,393 | 3,426 | 5,568 | 10,298 | 8,515 | 4,137 | 4,013 | 11,524 | NA | 11,016 |

NA = not applicable.
